# Selections and Abstracts

**Published:** 1900-03

**Authors:** 


### SELECTIONS AND ABSTRACTS.

---

### The Reflex Symptoms of Retroversion of the Uterus.

By A. Lapthorn Smith, B.A., M.D., M.R. C.S., Eng.

Fellow of the British and American Gynecological Societies; Gynecologist to the Montreal Dispensary.

Although I have operated upon and, with a few exceptions, cured one hundred and thirty-five cases of retroversion with adhesions by performing ventrofixation, and eighty cases of retroversion without adhesion by Alexander's operation of shortening the round ligaments, I am not yet satisfied, for I am convinced that there are yet several hundreds of women in this province alone who are suffering from this disease with its numerous symptoms. These women could be cured almost without risk by one or the other of these two operations. I say almost without risk, because when there are adhesions and the abdomen has to be opened, and perhaps a diseased ovary has to be removed, even then there is only a risk of about one per cent. While, if there are no adhesions and we can put up the uterus with the bimanual manipulations or by means of the sound and hold it up by shortening the round ligaments, this operation has been so perfected that there is absolutely no danger at all. During the first years after it was introduced there were many hernias and sometimes a death, but these accidents are no longer to be feared in the hands of operators of experience and with our present rigorous asepsis.

In this short paper I propose only to call attention to the symptoms, and especially the more obscure ones, by which this condition can be recognized. We must suspect its presence, and then search for it by a vaginal, preferably a bimanual examination, whenever a woman comes to us with the following symptoms:

1. Bladder troubles manifested by frequency of micturition caused by the pressure of the cervix against the neck of the bladder. When the fundus goes backwards the cervix, as a rule, points forwards.

2. Troubles in the rectum, either pain during defecation or obstinate constipation even when the patient's bowels have been made liquid by means of purgatives, the uterus acting as a rubber valve, the more the woman bears down the tighter it closes. Sometimes there is dysentery or rectal tenesmus due to the pressure of the heavy fundus on the rectum which ends by ulcerating it; even when there is nothing in it the patient feels as if the bowel was full. This is one of the obscure symptoms and must be searched for, as the woman will, as a rule, tell us that she is constipated, and it is only by questioning her that we will ascertain that her movements are liquid. When this condition of the stools is present, we will surely find either a stricture of the rectum or obstruction from the retroverted fundus.

3. Disorders of the brain and nervous system. The great sympathetic nerve, contrary to the cerebro-spinal system, has its brain at the lower end of the spine, just about the place where the retroverted fundus will lie upon it, so that with every movement the woman makes, the great sympathetic receives a blow or impression which is conveyed to the brain, causing headaches and neuralgias in other organs, the heart, lungs, liver, stomach and bowels. More than once I have had patients who vomited constantly until the cause was discovered in a retroverted uterus, the vomiting stopping at once on replacing the displaced organ.

4. Disorders of intelligence. When the irritation has lasted a long time the nutrition of the brain suffers seriously, and the patient may even lose her reason. I can recall at least a dozen cases in which the women themselves told me that they were ashamed of themselves for being so disagreeable to their husbands; the kinder their husbands were to them the worse they treated them. Two months ago a lady came to me from British Columbia to consult me on account of nervous attacks. She did not know the cause of them, but she assured me that if I did not discover it, and cure her, her husband would leave her, so disagreeable towards him had she become. Her father assured me that she had formerly been of a sweet and gentle disposition, and that a great change had taken place in her since a few years. She presented such a pale and worried appearance that I at once suspected that she had a retroversion, and, on examining her, my suspicions proved to be well founded; there was a large and heavy uterus with the fundus lying in the hollow of the sacrum. A few days later the round ligaments were shortened, and, after three weeks, she assured me that she felt quite differently. Another woman, whose history lies before me as I write, stated that for nine months previously, she had been having strange ideas; for instance, she had an almost irresistible impulse to throw herself out of her bedroom window; she was, therefore, afraid to go up to her room unless some one went with her. Also, when she had a knife in her hand there was a great temptation to drive it into her heart or to cut her throat with it, so that she had to throw it down and run away from it. I at once suspected a retroversion, looked for it and found it. As it was firmly fixed, I had to open the abdomen and do ventrofixation. The very next day on my visit to the hospital she expressed her gratitude, saying: "Whatever you did you have removed that cloud from my brain; my trouble has gone." On asking her if she had much pain from the operation, she replied that "That was of no account compared with the sadness which before had overpowered her."

5. Dyspareunia and sterility. Of the two hundred and fifteen women who were operated on for displacements, about three-quarters, or about one hundred and fifty, were married, and nearly all of these complained of pain on coitus. This is not surprising when we remember that the uterus is directly in the road of the male organ, and only about an inch or two from the vulva. Moreover, it is swollen and exceedingly tender, several patients having described their pain as being similar to the throbbing of a whitlow. When a woman is irritable and angry with every one, it is easy to understand that she will not be pleased with her husband for causing her such severe pain as coitus under these conditions implies. As for the sterility, about twenty babies have followed the eighty Alexander operations, but only two or three have followed the much larger number of ventrofixations, this being due to the fact that when there is fixation the tubes are always diseased. I did not think that the forwards or backward position of the cervix was of so much consequence to fecundation until I had seen pregnancy follow immediately, and without any other treatment, in women, who had been married many years without having children, after placing the cervix in its proper place. One young woman who had been having illicit intercourse during several years without any consequences because she had a retroversion began to suffer so much pain that her physician sent her to me for an Alexander. The operation succeeded so well that on her return to her lover she immediately became pregnant and had a normal delivery.

6. Symptoms due to the uterus, ovaries and tubes. Dysmenorrhœa and menorrhagia. Almost all these women suffered from dysmenorrhœa caused by the pronounced congestion of the genital organs. The blood is pumped into them by the arteries, but cannot get out by the veins because they are twisted and compressed.

The endometrium becomes varicose and swollen, causing both a painful obstruction to the escape of the blood and a profuse flow. The ovaries become so inflamed that ovulation causes excruciating pain.

7. Miscarriages. When a woman has had several miscarriages at the third month which are not due to syphilis, we will almost surely find on examining her that she has a retroversion. A few months ago I was called by Dr. Grant Stewart to a case of retention of urine due to retroversion of the pregnant uterus. She was suffering terribly, and a miscarriage would soon have come on; her physician tried to get the uterus up, but found it firmly wedged below the promontory of the sacrum. Although I placed her in the genu-pectoral position, it was only after a quarter of an hour's firm pressure with my finger that I was able to dislodge it from between the utero-sacral ligaments. I need hardly add that all her symptoms disappeared the moment the uterus was replaced. Another woman, who was sent to me by Dr. King of Compton, was pregnant about three months, and the fundus filled the pelvis, the uterus being bent on itself and the cervix being flattened against the symphisis pubis. It was impossible to get the fundus up without opening the abdomen, which I did, and then performed a ventrofixation. She had a normal confinement, and the womb has not fallen since.

We used to think that it was a sort of moral rape to make a vaginal examination of an unmarried woman, but now we know that virgins suffer from displacements quite often, and that their suffering cannot be remedied without removing the cause. While writing these pages, a single woman of thirty came to my office complaining of dysentery for which she had consulted me three years ago, at which time I did not examine her, and as I did not do her any good she left me and passed through the care of several other physicians without any benefit. This time, however, she insisted that I would examine her, as she felt sure that her womb was the cause of her trouble. And she was right, for on making a vaginal examination I found it completely turned. To-morrow morning I have to operate on another girl who consulted me for dysmenorrhea, which compels her to remain in bed one day every month, and her period lasts seven days, and is profuse. She was so tender that it was impossible for me to examine her without an anesthetic. With one I ascertained that she had a retroversion, which was easily replaced with the sound. I intend to dilate, curette and shorten the round ligaments at one sitting.

In conclusion, I would advise every family physician to make an examination, either with or without an anesthetic, so as to assure himself whether there is a retroversion of the uterus, not only in those cases in which the symptoms point directly to the uterus, but also when there are reflex symptoms which might possibly be due to this cause.—*Canadian Medical Record.*

---

### TREATMENT OF PUERPERAL FEVER.

At the sixty-seventh annual meeting of the British Medical Association, in the course of a discussion on this subject, Professor Spencer spoke as follows:

"The most important part of the treatment of puerperal fever consists in the prophylaxis. Although much is still unsettled in the scientific aspect of the subject, the value of prophylactic treatment has been conclusively proven by abundant clinical experience. Here I venture to recommend again the routine examination of every patient to the end of pregnancy. Only by this means can contracted pelvis and tumors and purulent foci be discovered and measures taken to prevent the dangers to which they may give rise during labor. It is most important to avoid all unnecessary injury during labor, by too frequent examinations, by unnecessary forceps operations, or on the other hand, by allowing the labor to be unduly prolonged. Abdominal palpation should to a great extent take the place of vaginal examination.

"Careful management of the third stage of labor is of the highest importance, and particularly is it necessary to make sure that no placenta or membranes or clots are allowed to remain in the uterine cavity.

"An equally important feature in the management of the labor is the thorough disinfection of the hands and vulva. The experiments of Döderlein have shown that in normal cases vaginal injections, so far from preventing the growth of micro-organisms, rather favor it, but in every case of labor the vulva should be washed with soap and water, rinsed with water, and swabbed with 1-in-1000 perchloride of mercury. The hands (of which the nails should always be short), wrists, and forearms should be thoroughly scrubbed with soap and hot water, rinsed with water, and soaked in 1-in-1000 perchloride of mercury solution (in place of this some prefer alcohol). The safest lubricant is a solution of perchloride of mercury in glycerin of the strength of 1-in-1000; instruments, if kept bright, require no lubricant, but should be immersed in a solution of carbolic acid (1-in-20). All instruments should be of metal or glass, and should be disinfected by boiling. I would add a word on aseptic clothing. The ideal clothing is white washable material. In private practice this is not always available, and a compromise may be effected between it and the ordinary black coat by drawing up the sleeves and pinning round the forearm a clean linen towel. In this matter even old Smellie, one hundred and fifty years ago, was ahead of us, for he used to slip a nightgown over his clothing, and no doubt he found it very convenient when he took to his bed, as seems to have been his custom when tired by a difficult labor.

"The question here presents itself as to whether it is necessary for a doctor who has attended a case of puerperal fever to abstain from practice for a time. Those who give the advice that it is necessary are generally those who are themselves engaged in examining septic cases. Mere abstention from practice will not prevent the carrying of infection when practice is resumed; thorough disinfection will immediately enable a practitioner to attend another lying-in woman with safety.

"As regards the actual treatment of puerperal fever, when it has arisen, it is necessary, in the first place, that a thorough examination of the patient and of the uterus be made. The necessity of examining the uterus in these cases has been insisted upon by Dr. Cullingworth; and although my experience does not coincide with his as to the frequency with which portions of the placenta are found in the uterus in these cases, and the researches of Bumm show that more harm than good is likely to result from removal of minute adherent particles, yet I am convinced of the advisability of carrying out an intra-uterine examination in nearly every case. In many cases apparently almost hopeless, as a result of this examination I have removed putrid portions of placenta with recovery of the patient, and in three cases I have removed fibroid tumors by enucleation with an equally satisfactory result.

"In order to avoid infecting the hands in examining a septic case I can strongly recommend the wearing of rubber gloves, which I have used for the last four or five years in examining syphilitic and septic cases and in extraperitoneal operations. The gloves I have used are the ordinary post-mortem gloves, but the thin rubber gloves which have lately been made in Germany [now made in Akron, Ohio—ED.] are far preferable for the purposes of examination, as they scarcely interfere with tactile sensation, and cause less discomfort to the patient than the uncovered finger.

"Speaking generally, a septic substance found in the uterus should be removed, but there are some rare exceptions, of which I may mention strongly adherent pieces of placenta unattended by severe general symptoms (though often by high fever and foul discharge); these cases will often do better if the elimination of the substance be left to nature than if violent and generally ineffectual attempts be made to remove them. But, speaking generally, the uterus should be completely emptied by the finger, occasionally by large, blunt-ended forceps, and, I think, never by means of the curette after labor at full term.

"I have found post-mortem the uterus so soft that the finger and thumb could pulp the organ like a fatty liver, and I have known the finger to be forced through the wall of the puerperal uterus during life; how much greater is the danger associated with a sharp instrument like the curette!

"After emptying the uterus, in all cases where there is a foul discharge I usually irrigate the uterus once with a solution of iodine (one drachm of tincture of iodine to the pint), or boracic acid, or by a large quantity of salt solution. Continuous irrigation of the uterus (first recommended by Schücking in 1877) is certainly useful in cases where some adherent tissue has to be left behind. Nevertheless, it is very irksome to the patient, and the tube is apt by its pressure to cause troublesome sores, and has even been known to perforate the uterus (see Pinard and Wallich), so that I have almost entirely abandoned continuous irrigation and even oft-repeated irrigation, unless beneficial results of the first irrigation are marked.

"The best intra-uterine tube is, I think, Budin's catheter made of glass. In order to clean the septic endometrium Upshur has advised the employment of peroxide of hydrogen, which is very useful in cleaning up sloughy wounds, though I have no experience of its use in the uterus.

"All forms of intra-uterine irrigation and even vaginal irrigation are attended with some risk—risk of injury by the antiseptic employed, risk of perforation of the uterus, and particularly the unavoidable risk of embolism, of which I have seen two cases within the last twelve months, which gave rise to the greatest anxiety for a few hours after its occurrence, though somewhat to our surprise they both recovered. In order to drain the endometrium iodoform gauze has been employed; this also is not unattended with the risk of air embolism. I rarely employ it for draining the puerperal uterus, inasmuch as discharges are apt to be more copious than the iodoform gauze can well deal with.

"An interesting modification of the gauze drainage is that proposed by Carossa—namely, the introduction of a catheter into the uterus, packing absorbent cotton or gauze around the catheter, and the introduction through the catheter of three teaspoonfuls of 25 per cent. alcohol every hour.

"The treatment of general septic peritonitis has been much discussed of late years. It has been proposed to open the peritoneum either by the abdomen or the vagina, and even to remove the whole uterus. A number of successful cases of both methods of treatment have been published. Prior has advised the opening of Douglas's pouch and isolating the uterus from the general peritoneal cavity by means of iodoform gauze. The difficulty about the treatment of diffuse peritonitis lies in the uncertainty of the diagnosis until the condition is well advanced. In cases where there is clearly septic fluid in the peritoneum there is no doubt that operation is indicated. For my own part I have always waited until there has been evidences of localization of the fluid, and then have opened the collection either through the abdominal wall or through Douglas's pouch. The milder forms of localized sepsis (perimetritis, parametritis, and phlebitis) are treated on the general principles of rest, hot and anodyne applications, etc.

"With regard to the removal of the uterus in puerperal sepsis, it is satisfactory to learn that a patient can survive this operation, but the cases for which it is suitable appear to me to be very few, such as a uterus infected by carcinoma or by a fibroid which cannot be enucleated.

"Other methods of remotely influencing puerperal fever are the nuclein treatment, which aims at producing an artificial leucocytosis, the fixation abscess of Fochier produced by the injection of turpentine into the cellular tissue (von Zwiccicki, Thierry), saline infusion into the cellular tissue or its administration by mouth (Zwiccicki) or rectum, intravenous injections of antiseptics (v. Kezmarszky), and ligature of infected veins. The last two methods do not commend themselves to my judgment, and the last in the few cases in which it has been tried has been unsuccessful.

"With regard to the medicinal treatment of puerperal fever there is not much to be said. Though in mild forms of infection aperients sometimes do good, they are not unattended with danger in the severe forms, being apt to set up diarrhea, which is very difficult to control. I know of no drug which beneficially influences the temperature in puerperal fever, and I would most strongly deprecate the use of antipyretics for this purpose; though they will lower the fever, they also reduce the strength of the patient. Quinine may be given as a tonic; as an antipyretic I believe it is useless. I have given eighty grains in a few hours without producing any effect upon the fever.

"Puerperal fever patients need tonics and stimulants (alcohol, strychnine, digitalis, etc.), but the depressant antipyretics should, in my opinion, never be employed; cold packing is a much better method of reducing the temperature.

"Fresh air is very necessary in the treatment of septicemia. The beneficial effect of the removal of a poor patient from the close, hot atmosphere of the house to a well ventilated hospital ward is often very striking.

"With regard to the diet, the administration of food at regular intervals in quantity as great as the patient can take is, in my opinion, the most important part of the treatment, and alcohol may be given in large quantities with benefit. Where vomiting occurs rectal feeding with peptones should be employed."—*British Medical Journal, October 14, 1899.*

---

### CRANIECTOMY FOR MICROCEPHALIC IDIOCY.

W. J. Wilson, M.D. (*Canadian Journal of Medicine and Surgery*, 1899, vi., 425), in a brief article on "Craniectomy for Microcephalic Idiocy," says there are so many conditions which, in microcephalus with idiocy, may be present either as a cause or simply in association—conditions which during life it may be impossible to diagnose—that the question whether we should operate in any given case becomes a matter of very serious consideration. There can be no doubt that premature ossification does take place, that fontanelles may be closed and the sutures ossified at birth, but whether this is a cause of the microcephaly, as was thought to be the case by Lannelongue, or a result of arrest of brain development is still a debatable question. When gross cerebral lesions, such as extensive sclerosis exist, the chances for improvement cannot be good. Operations on the skull for microcephalic idiocy will not take a permanent place in our treatment until a large number of cases have been observed, and we are able to have judged better just what conditions may or may nôt be improved by operation. It is with this object in view that the following case is presented: Henry R., aged five years. Family history good. No idiocy, deformities or insanity in the family of either parent. No history of tuberculosis or syphilis. One sister died from laryngeal diphtheria. Has two healthy brothers. There is a difference in the age between Henry and the next elder child of fourteen years. During these fourteen years there were no miscarriages. One since. Parents both healthy and strong; grandparents on both sides lived between seventy and eighty years. Other members of the family have average-size square heads. Mother had no frights and no thoughts of trouble during gestation. At birth baby weighed seven pounds, was plump and fat, but his head was very small. His mother noticed there were no openings in his head.

Dr. Webster, who saw the child when six months old, says at that time there was no trace of fontanelles. He was a small feeder. Was nursed on the breast for fifteen months, but did not thrive well. At sixteen months he weighed fifteen pounds, was apparently healthy, but did not grow like other children. He was three years old before he could stand alone. His ankles were weak and turned in. Sight and hearing normal so far as known. In walking he bent forward almost to a right angle. He was restless and cried a great deal, was a bad sleeper, waking frequently during the night. When four years old began taking thyroid tablets. He began with one five-grain tablet per day, and by the end of nine months was taking four tablets or twenty grains of P. D. & Co.'s thyroid extract per day.

During this time his legs improved very much in strength and became straighter at the ankles, but body remained bent over in walking. Five months ago he was admitted under the author's care at the Western Hospital. At this time he could only say one word—mamma—and this he used for every person. He would not play with his toys, was very restless and rubbed his head a great deal. He was then five years old, and it was noticed he was cutting his lower central incisor teeth. His first dentition began at the fifth month. His disposition was affectionate, but at times he became vicious towards other children. The cranial measurements, after hair was removed, were: Circumference at tip of ears, $14\frac{1}{4}$ inches; over vertex from meatus to meatus, $9\frac{1}{4}$ inches; diagonally over frontal bone from meatus to meatus, $8\frac{3}{4}$ inches; diagonally over occiput from meatus to meatus, 9 inches. Early in April an incision was made on one side of the sagittal suture and about one inch from it. At the first incision a piece of bone one by two inches was removed. This was followed by very little shock, and in about three weeks a similar incision was made on the other side of the head. These two incisions were extended to about four inches by one inch on either side of the sagittal suture, and at periods of two or three weeks apart, making in all, four operations.

The skull was first opened with a trephine, and the opening then extended by the aid of punch forceps. The periosteum was pushed to one side, but not removed, and the dura mater was not opened. There was some bulging of the dura at each operation. The skull was not thick, but so dense it took a very hard pressure of the forceps to cut it through. There was not much hemorrhage, but it was remarked there was an increased amount at each operation. The bone lifted easily from the dura except during the last operation, which was on the posterior part of right incision, and here it was attached more firmly. No other conditions were noted. Shock was very slight after operation, and next morning he wanted to sit up in bed as usual. Some improvement was made from the first, He became easier to manage, and played more with his toys. He has learned a great many words and short sentences, and is learning more daily. His disposition is milder, he sleeps all night through, and has quit, for the most part, movements of his hands. which before operation were very marked. While he does not always stand erect or walk without some bend forward, his gait is vastly improved and he stands erect most of the time. He has had no special training since operation other than by his mother, so that training does not account for his improvement. Henry has always been quite musical and picks up tunes readily. He did this before operation.

When he came into hospital his circulation was bad, his hands cold, blue and damp. This was attributed to the use of the thyroid, and after it was discontinued his circulation improved.

The conditions in this case which decided for operation were (1) early ossification of sutures and fontanelles and early dentition; (2) absence in so far as could be discovered of gross brain lesions.—*Gaillard's Medical Journal.*

---

### NOTES ON THE USE OF SUPRARENAL EXTRACT IN RHINOLOGY.

Yearsley contributes these notes to the *British Medical Journal* of October 14, 1899. He begins by pointing out that suprarenal extract as a vasoconstrictor in rhinology has been in use for some little time. Most of the investigations have been made in America, and the paper of Newcomb, of New York, gives a good review of the subject. The experiments on living animals of Schäfer, Oliver, Cybulski, and Szymonowicz showed that the results of intravenous injections of extract of the suprarenal medulla are as follows:

1. Extreme contraction of the arteries, peripheral in origin.
2. Rapid rise of blood-pressure, despite powerful cardiac inhibition and augmented by section of the vagi or paralysis of the cardiac inhibitory nerves by atropine.
3. Pronounced central nerve stimulation.
4. Great acceleration and augmentation of the auricular and ventricular contraction after section of the vagi.
5. Only slight affection of respiration, which becomes more shallow.

As a therapeutic agent in diseases of the nose suprarenal extract was first used in America by Bates, of New York. Later Swain, in a paper before the Congress of the American Laryngological Association at Brooklyn in May, 1898, stated that the aqueous extract of suprarenal gland was a powerful local vasoconstrictor and contractor of erectile tissue, and could be used in very considerable amounts without dangerous or deleterious effects, local or constitutional. Newcomb says: "It is really remarkable to notice how the redundant tissue so often met with in the nose will shrivel and become pallid when the solution is applied." Mullen, of Texas, gives the notes of seven cases (of turbinectomies, removal of spurs, and operation for deviation of the septum) in which he used the extract. In his opinion suprarenal extract is a valuable aid to nasal surgery as a hemostatic and analgesic. He used a solution of five grains to the drachm. Lermitte and Peters have also recommended this drug.

The chief difficulty which Yearsley found in using the drug lay in obtaining a solution at once reliable and capable of keeping. Lederman, of New York, has recommended a glycerin watery solution, and for a short time Yearsley used a preparation made after his directions. In his first cases the solution was 5 per cent., made by dissolving a 5-grain tablet in 100 minims of sterilized distilled water. Later he found it better to use a stronger solution of 5 grains to the drachm. Pledgets of cotton-wool soaked in the solution are adjusted by forceps and probe. In no instance has a spray been made use of.

The extract was used to ascertain its effect upon the normal nasal tissues in a few cases in which there was practically no obstruction to natural nasal respiration. In all, after the introduction of a 5-per-cent solution from five to fifteen minutes, the turbinal bodies became shriveled and pallid, the ischemia being so marked as to give a clear view of the posterior pharyngeal wall. All patients experienced a sense of dryness in the nasal chambers. The effect lasted for periods varying from half an hour to two hours. In one or two instances slight smarting was complained of, but this was never a prominent symptom.

The extract was used in some forty cases as an aid to diagnosis, and the marked ischemia it caused was most useful in revealing causes of obstruction. Turbinal hypertrophies, growths situated posteriorly or hidden by turbinal turgescence, posterior spurs, etc., became easily visible. In a case in which the inferior turbinal was very edematous and pressing upon the septum, the swelling was reduced by one half, so bringing to light a single polypus growing down into the inferior meatus, and a large posterior spur.

Yearsley has made trial of the drug as a hemostatic in about thirty instances, using it in some cases more than once. The operations in which it was employed were: (1) Anterior turbinectomy; (2) removal of polypi; (3) removal of spurs; (4) division of adhesions; (5) removal of a foreign body. Besides the increased room which the ischemia affords, hemorrhage is greatly lessened. Operations usually attended with a fair amount of bleeding become almost bloodless. The advantage of its use in nasal operations is therefore threefold; there is greater room by reason of the ischemia, the loss of blood is far less, and the field of operation is not obscured by copious hemorrhage. In no case has he found any postoperative hemorrhage, such as sometimes follows the use of cocain. The anesthetic used was a 10-per-cent. solution of eucain. In no case was the extract of suprarenal gland found to have any anesthetic effects.

Solis Cohen found that the extract of suprarenal capsule controlled the symptoms of hay-fever in his own case. He first used a glycerin extract of sheep's suprarenal capsule, freshly prepared in doses of from 15 drops to 1 teaspoonful thrice daily. Later, he found it necessary to either increase the dose or to repeat it more frequently. As a tendency to nausea was produced with this extract, he substituted for it tablets. On the average he took five 5-grain tablets daily, the last one being taken at bedtime, with the result that the night passed without his suffering from any of the usual symptoms.—*Therapeutic Gazette.*

---

### DIGITALIS IN THE TREATMENT OF HEART DISEASE.

BY DR. W. H. WASHBURN (*Merck's Archives*, I, No. 10, p. 416).

The action of digitalis, says the author, is upon muscular fiber, causing it to relax more slowly and to contract more quickly and perfectly. Its effects are not alone limited to the heart, but extend to the muscular system in general. Digitalis increases blood-pressure and converts the intermittent circulation in the capillaries into a steady flow. The increased blood-pressure lessens the rapidity of the heart's action, as well as does the action of the drug upon the vagus. The whole amount of blood in the arterial system is increased, and there is a corresponding lessening of the amount in the veins. This stimulates the absorption of serous exudates. The myocardium, like other tissues of the body, receives an increased blood-supply, and consequently its nutrition improves, thus favoring compensating hypertrophy. Digitalis is not contraindicated in aortic regurgitation and full pulse. The administration of digitalis is much hampered by fear of its cumulative effects. This is a real danger, as it is a drug that is absorbed slowly, and elimination is by no means rapid. In consequence we may have a chronic or acute intoxication if the doses are small and administered at short intervals. Chronic poisoning by digitalis results in contracted arterioles and in increase of resistance in the peripheral circulation. The coronary arteries, in common with other arterial trunks, share in this condition, and consequently, as a result of too free administration of the drug, the nutrition of the myocardium is lessened, with a consequent aggravation of the heart-failure.

The dose of digitalis that will prove tonic to the heart is a moderate one; this dose must only be repeated after a sufficient interval. If the dose be excessive and the intervals between the doses are too short, unfavorable results may follow. A fair dose of general application is the use of one grain of the powdered leaves, or its equivalent, in any of the official preparations, administered once in twelve hours. This is a dose that can be continued for months or years without saturating the system. Such doses may not produce the immediate results which are often desired by the friends of patients, but under its administration a feeble, almost imperceptible, pulse gradually improves, with a corresponding amelioration of the general symptoms. If larger doses are employed, symptoms of saturation should be looked for, which is shown by a slowing of the pulse-rate, lessened diuresis, and more rarely diarrhea.—*Post-Graduate.*

---

### SELF-MEDICATION.

The habit of self-medication causes far more deaths every year in our country than the carnage inflicted on both sides during our recent war with Spain. Our gambling propensities as a nation are shown in the worst light when we gamble on our lives. The digestive tract appears to be a slot-machine, into which its possessor takes pleasure in thrusting any preparation with a euphonious name suggested for the "cure" of disease in newspapers, in circulars on our door-steps, on posters on the dead walls of cities, in the street-cars, on the fences along the line of travel, yea! on the mountain-tops, to delude the healthy or seeker of health from enjoying the advantages of such a salutary region. A morbid hysterical condition often results in many people by reading and studying flaming and picturesque notices regarding their nerves, livers, hearts, kidneys, etc. Laboring under the delusion of being ill, thousands will unnecessarily take drugs without consulting a physician—try their luck in "cures" as they would in cards at the faro-table—and become dyspeptics, neurasthenics, and inebriates in consequence. Instead of obtaining the best remedies and advice from those qualified by education and experience to bestow them, people will pour drugs of which they know nothing into a body of which they know less, and will try, and keep on trying their luck as long as the pleasing advertisements appear, to be changed only when a more alluring picture and catchy testimonial of a "sure cure" presents itself—4,11,44, you pay your money and you take your choice. A veritable lottery on life! The great lottery drawings of the world will give a list of the few big prizes that are won, but the names of the thousands and thousands who did not succeed in getting anything are not intended to be known by the emotional and foolish creatures who invest their money in such gambling schemes. And so it is with patent medicines. The multitude whose constitutions are really injured, or who received no benefit whatever from those nostrums, are never heard of. "Foolery, sir, does walk about the orb like the sun; it shines everywhere." How fortunate it would be for humanity if the tons of headache powders, the barrels of blood-purifiers, and the hogsheads of liver pills were thrown into the sea rather than into the stomachs of people by men who scarcely know the name of a bone or muscle or the function of a single organ of the body!—WM. B. DOHERTY, M.D., Louisville, Ky.—*New York Lancet.*

---

### TONSILLITIS AND RHEUMATISM.

From the fact that tonsillitis often occurs as a precursor of acute arthritis, that it is occasionally undoubtedly followed by acute endocarditis and is sometimes benefited by the administration of salicylates, many are inclined to regard it as of rheumatic origin in many cases. Dr. Packard, of Philadelphia, in a recent article in the *American Journal of the Medical Sciences,* reports several cases of endocarditis and arthritis complicating acute angina. He is disposed to regard these complications not as manifestations of acute rheumatism, but rather as cases of irritation of serous surfaces by germs or their toxins which enter the general circulation from the inflamed tonsils. He cites a case of endocarditis which died of broncho-pneumonia, reported by Charrin, in which at autopsy the staphylococcus pyogenes aureus was found both in the tonsils and in the vegetations on the valves of the pulmonary artery. In the cases of endocarditis associated with tonsillitis reported, Packard was unable to find any evidence of articular trouble preceding the attack and in some cases he knew the heart to have been normal previous to it. If we designate these cases rheumatism, it is apparent that tonsillitis stands as a cause rather than as an effect of the former disease. Indeed, this latter view is consistent with the so-called germ-hypothesis as to the origin of acute articular rheumatism in contradistinction to the metabolic and nervous hypotheses. This might lead us to the view that acute rheumatism is not due to one specific organism but is rather a form of septic intoxication or septic infection due to the absorption of the products of several species of micro-organisms or the absorption of the germs themselves in some cases. That angina is not always present in rheumatism is true, but the same process of inflammation and absorption may possibly go on elsewhere,—in the gastro-intestinal tract for instance.—*Pediatrics.*

---

### THE RELATION OF MIGRAINE TO EPILEPSY.

W. G. Spiller (*American Journal of the Medical Sciences,* December, 1899), from his observations on this subject, draws the following conclusions:

1. Attacks of migraine occur associated with nausea and vomiting; this form is known as simple migraine, and usually remains unaltered during the life of the patient.

2. In other cases visual disturbances (hemianopsia, scintillating scotoma, amaurosis, etc.) are associated with the migraine, and the disease is then known as ophthalmic migraine.

3. When paralysis of the ocular muscles occurs with the migraine, the disease is described as ophthalmoplegic migraine.

4. Migraine, especially the ophthalmic form, is related to epilepsy, and the attacks of migraine may precede for many years the convulsive attacks of epilepsy, although in most cases of migraine no convulsions are ever detected.

5. In some cases epilepsy appears in the form of one or more of the disturbances seen occasionally with migraine, and later, even after many years, convulsions develop. The disease may be epilepsy from the beginning. It matters little, with our uncertain knowledge of the pathology of the two diseases, whether we regard these as abortive cases (forms frustes) of migraine that later become associated with epilepsy, or as abortive forms of epilepsy (sensory epilepsy), in which the convulsions later become apparent, provided we recognize a relation between some forms of migraine and epilepsy.—*Gaillard's Med. Journal.*

---

### PAIN–ENDURING ANIMALS.

The manner in which animals and birds endure pain, says a writer in a popular magazine, should awaken the sympathy of all thinking people. Horses in battle furnish a striking example of this power of endurance. After the first stinging pain is felt they make no sound, but bear it with mute wondering endurance, and when in the silence of the night a groan comes from the battlefield it is because of loneliness—the craving for human companionship which is so necessary to once domesticated animals. A dog will go for days with a broken leg without complaint, but the pleading, wistful look would attract attention from any one not totally blind to all sensibility. A cat wounded by stick or stone, or caught in some trap from which it has either gnawed or pulled its way, will crawl to some quiet, out-of-the-way place and endure silent agony which we could not endure. Cattle will meet the thrust of the butcher's knife without a sound. The wild dove with shot from the hunter's gun burning in its tender flesh will fly to some high bough or lie upon the ground and die, and no sound will be heard, save the dripping of blood upon the leaves. The stricken deer will speed to some thick wood and there in pitiful submission await the end. The eagle stricken in high air will struggle to the last, but there will be no sound of pain, and the proud, defiant look will not leave the eyes until the lids close over them and shut out the sunlight they loved so well.—*Western Druggist.*

### THE DIAGNOSIS OF TUMORS OF THE HEAD OF THE PANCREAS.

*Zoja* (*Il Policlinico*, August 1, 1899), in an attempt to arrive at certain clinical criteria for the elucidation of the above subject, is driven to the conclusion that as far as primary carcinoma of the head of the pancreas is concerned there is no proper or constant symptomatology; the symptoms vary so much according to the position and extension of the tumor. The cases may be divided into three chief types: (1) where the tumor takes a postero-inferior direction, causing compression of the vena cava, implicating the right suprarenal capsule and the retroperitoneal glands; (2) where it takes an upward direction, causing symptoms of occluded bile duct or of pyloric stenosis; (3) and (perhaps the most common) where development takes place to the right, giving rise to occluded bile or pancreatic ducts. A complete knowledge of the effects likely to be produced by the occlusion of these ducts is important. As regards individual symptoms, jaundice is by no means constant. The condition of the gall bladder affords no certain criterion. The changes which occur in the liver are either due to metastatic growths or to the effects of prolonged occlusion of bile ducts. Tumor may be felt; it depends on the stiuation, etc. Pain is not a constant symptom; melena or hematemesis is a fairly common termination. The author never observed glycosuria in his cases, and attaches no importance to the search for indican. There is an extensive bibliography.—*Brit. Med.*

---

### AN INTERESTING EXPERIMENT.

At a meeting of the Western Surgical and Gynecological Society the last week of December, an exceedingly interesting incident took place. In 1870 Messrs. William R. Warner & Co. filled an order for pills for Messrs. Chilcote & Cook, of Washington, Iowa. One of the bottles being still on hand, it was forwarded to Dr. Milton McCarthy, of Des Moines, Iowa, to test the solubility of the pills before the above mentioned society. The following correspondence explains the results fully:

WASHINGTON, IOWA, Dec. 23, 1899.

*W. W. McCarthy, M.D., Des Moines, Iowa:*

DEAR SIR:—Having some pills manufactured for us by Wm. R. Warner

& Co., in the latter part of 1870 (at the request of Dr. W. E. Fraser, then a practicing physician in this place), which are still in good condition, and thinking it might be of interest to the physicians who meet in Des Moines during the coming week, to see a pill made twenty-nine years ago, in as good condition as these are, we forward them for your and their inspection. A manufacturer who places on the market goods which stand the test these have is worthy of commendation.

Yours truly,

CHILCOTE & COOK.

State of Iowa, Washington County. Subscribed and sworn to before me this 23d day of December, 1899.

L. E. LATTA, Notary Public.

DES MOINES, IOWA, Jan. 1, 1900.

*Wm. R. Warner & Co.:*

I am pleased to inform you that the pills which were made by your firm in 1870, and sent to me by Chilcote & Cook, of Washington, Iowa, were tested as to their solubility at the meeting of the "Western Surgical and Gynecological Society," held in our city last week. They were readily soluble in water at a temperature of 100 degrees, and were completely disintegrated at the expiration of fourteen and one-half minutes.

Respectfully,

WILTON MCCARTHY, M.D.

Messrs. Warner & Co. have had on exhibition since 1873, a mahogany case filled with sugar-coated pills. After a quarter of a century they do not show the effects of age, but are as good as when first made, which means they are perfect pills.

---

### SEROTHERAPY AND ORGANOTHERAPY AMONG ANCIENT GREEKS.

Professor Lambadarios of Athens (*Greece Med.*, October), (*Jour. A.M.A.*) has written an interesting work in which he maintains that our most modern methods of therapeutics were foreshadowed by the ancients. King Mithridates he calls the father of artificial immunization and of serotherapy, as he experimented by taking a small amount of poison each day and thus rendering himself refractory to attempts to poison him. He also concocted an antidote which he took before or with the poison, believing that its action would be annulled by the antidote. It was made of every poison known at that date, including opium, combined with aromatic substances. He became so expert by experiments on himself and condemned criminals, that physicians applied to him as to a modern Pasteur institute. He also invented serotherapy, in his method of protecting against the bite of venomous serpents, by adding to his antidote the blood of animals fed on vipers. He selected for this purpose geese from the Black Sea, considering them especially refractory to the venom of vipers, as they are accustomed to feed on them. Pliny and others mention the fact that these geese eat vipers. Dioscorides treated hydrophobia by having the subject drink the blood and eat the cooked liver of the dog that had bitten him, and mentions a number of cures thus effected, among them, two physicians.—*Columbus Medical Journal.*

---

### THE TREATMENT OF DIGITALIS POISONING; EXPERIMENTAL INVESTIGATIONS.

Drs. J. J. Taylor and C. R. Marshall (*British Medical Journal*, November 4th) say: The treatment of digitalis poisoning must be symptomatic. We know of no antidote, in any true sense of the word, to this drug. Nor do we think we shall ever know of one. Digitalis enters very slowly into the tissue metabolism; it produces distinct structural changes, and it is slowly eliminated. It therefore possesses a most unfortunate character for action of the antidote. Apparently all that we can do is to withdraw the drug if it is being given medicinally, to clear the alimentary canal of any digitalis it may contain, and increase the rapidity of elimination by diluents; to allay sickness, to reduce arterial tension when high, procure sleep if necessary, and treat every symptom as it arises. In other words, the condition must be treated as if it were, which it is, an ill understood disease. For the reduction of the arterial tension nitroglycerine, or an ally, is the best remedy; but with the low blood pressure, which is not infrequently present in digitalis poisoning, these substances are useless. In such cases small doses of alcohol, insufficient to produce vasodilatation, would probably prove of greater service.—*Medical Record.*

---

**This Journal and Atlanta Semi-weekly Journal one year for $1 00 cash in advance. No checks.**

### ANTS IN SURGERY.

The *Pacific Record of Medicine and Surgery* quotes an interesting article from a Paris journal upon this subject. The process is briefly described as follows:

According to the "Entomologist" the Greek barbers of Smyrna apply the ants in the following manner: The edges of the wound having been approximated with the fingers of the left hand, the ant is held by means of pinchers and brought in contact with the wound, which it soon seizes and pierces through and through with its strong mandibulæ, thus holding the lips of the wound in apposition. The barber then severs the head from the body, leaving the former in place. The same process is employed with a number of ants until the entire wound is sutured. The heads are left in place for four days, when, if union has occurred, they are removed.

This method is said to have been used in very ancient times, and is mentioned in a French work as recent as 1845. Wounds of the intestines are known to have been sutured in this way.

---

### BATHS FOR INFANTS.

Dr. A. Jacobi teaches that while during the early days of an infant, a cold or even cool bath should not be given, yet after a few months, and by carefully graduating down the temperature of the water, a cool or cold bath can, and should, be used for infants, especially during the hot season. He believes that cold bathing promotes a very strong and healthy resistance to diseases, especially the enervating diseases of hot weather.—*Clinical Recorder*.

---

ACCORDING to the report of *The Lancet's* special commission to investigate the composition of cigarettes, there is no ground for the charge that phosphorus, opium, arsenic or mercury is used in making them.—*Medical Age*.

